# Freiburg Neuropathology Case Conference: A 43-year-old Patient Presenting with a Right-sided, Slowly Progressing, Painless Exophthalmos and Ptosis

**DOI:** 10.1007/s00062-023-01267-1

**Published:** 2023-02-20

**Authors:** B. Simon, E. Wogram, A. T. Camp, M. Prinz, H. Urbach, D. Erny, C. A. Taschner

**Affiliations:** 1 Department of Neuroradiology, University of Freiburg, Freiburg, Germany; 2 Department of Neuropathology, University of Freiburg, Freiburg, Germany; 3 Department of Ophthalmology, University of Freiburg, Freiburg, Germany; 4 Medical Centre—University of Freiburg, Faculty of Medicine, University of Freiburg, Breisacherstr. 64, 79106 Freiburg, Germany

**Keywords:** Primary orbital lymphoma, Orbital metastasis, Orbital rhabdomyosarcoma, Orbital schwannoma, Orbital solitary fibrous tumor

## Case Report

A 43-year-old male patient presented with a painless 5 mm exophthalmos of the right eye (OD), which had slowly developed over the past 12 months concurrently with a right-sided ptosis. He did not report any double vision.

On examination the palpebral aperture measured 5 mm OD and 10 mm for the left eye (OS). There was no swelling, redness or hyperthermia of the right eyelid. Ocular motility OD was largely unremarkable except for a slight elevation and abduction deficit on wide gaze excursion. Best-corrected visual acuity (BCVA) was 20/25 Snellen (0.8 dec) OD and 25/25 Snellen (1.0 dec) OS. Ophthalmological examination showed no evidence of optic nerve compression OD with no relative afferent pupillary defect, inconspicuous fundoscopy and optical coherence tomography of the retina and peripapillary retinal nerve fiber layer. A transnasal biopsy of the tumor previously performed at a peripheral hospital had shown unspecific results.

**Fig. 1:**
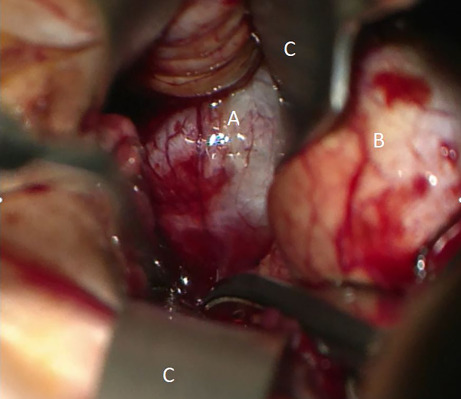
View of the operative site: tumor (*A*), right eyeball (*B*), spatulas (*C*)

The multidisciplinary tumor board recommended surgical excision of the tumor. Surgery was performed via a transconjunctival approach in general anesthesia using a surgical microscope as described previously [1]. Oculopression was performed preoperatively to lower eye pressure, facilitate lateral displacement of the globe within the orbit and thereby widen the surgical corridor. The conjunctiva was incised over 270° in the medial circumference. The superior and inferior rectus muscles were tethered with 4‑0 silk retraction sutures. The medial rectus muscle was detached from the globe. Another 4‑0 silk retraction suture at the muscle’s insertion point was used to displace the globe laterally. Into the medial quadrant of the parabulbar space, two narrow spatulas and one wide orbital spatula (Fig. 1) were inserted to form a triangular viewing channel. Spatula blades were inserted flat sides together and rotated into an orthogonal position once in place, thereby carefully displacing orbital structures sideways. In the depth of the parabulbar space, orbital fat was found. Its fine septa were opened with scissors at the tumor’s suspected location, revealing a homogeneous, white-colored, smoothly encapsulated tumor abutting the medial rectus muscle. A primary docking attempt with a cryostat was insufficient, so tumor grasping forceps were used for removal. Macroscopically, the tumor was a homogeneous, whitish, encapsulated, clearly circumscribed mass. Close microscopic inspection of the tumor bed confirmed complete removal and sufficient hemostasis after coagulation. The patient reported no pain or double vision 1 day after surgery. The BCVA OD was 20/50 Snellen (0.4 dec), likely due to swelling and irritation of the conjunctiva. No clinical signs of optic nerve compression were observed. After 2 days the patient was discharged from the hospital.

## Imaging

**Fig. 2:**
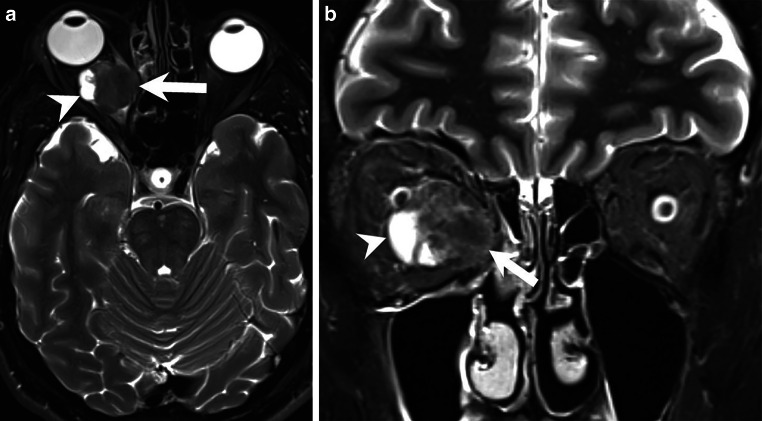
Axial (**a**) and coronal (**b**) T2-weighted images showed an intraorbital, intraconal mass (*arrows*) located medially of the optic nerve. Cystic components were presented on the lateral tumor margin (*arrowheads*). Note the right-sided exophthalmos on the axial view (**a**)

**Fig. 3:**
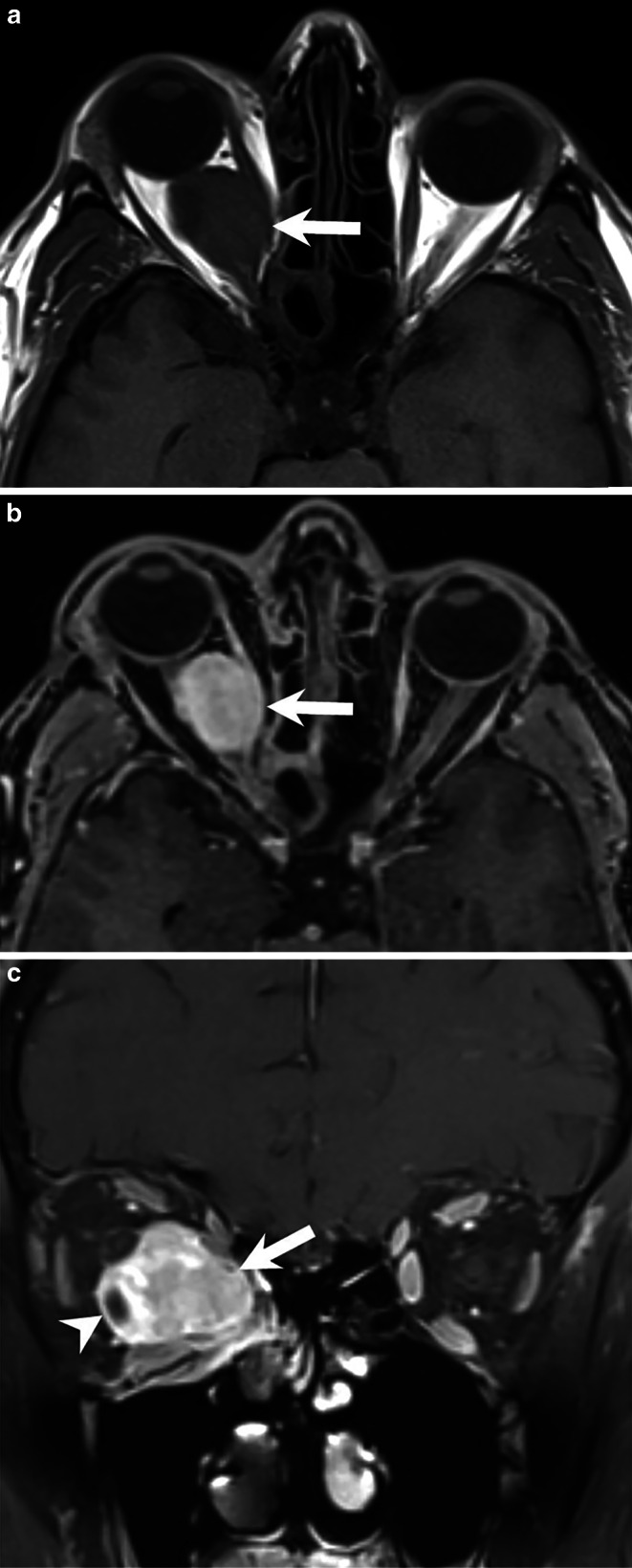
On T1-weighted axial images (**a**) without contrast the lesion (*arrow*) appeared isointense. On axial (**b**) and coronal (**c**) T1-weighted images MRI after administration of Gd the lesion showed homogeneous enhancement of contrast (*arrows*). The cystic portion of the tumor displayed a contrast-enhancing capsule (**c**, *arrowhead*)

Magnetic resonance (MR) imaging showed an intraorbital, intraconal space-occupying lesion located medially of the right optic nerve (Figs. 2 and 3, arrows). T2-weighted images (Fig. 2a, b, arrows) showed cystic components. Note the right-sided exophthalmos on axial images (Figs. 2a and 3a, b). On T1-weighted images the lesion appeared isointense (Fig. 3a, arrow). After administration of gadolinium (Gd) the lesion displayed distinct and homogeneous contrast enhancement (Fig. 3b, c, arrows). On diffusion-weighted images (B1000) the lesion showed no signs of restricted diffusion (not shown).

## Differential Diagnosis

### Orbital Lymphoma

Primary lymphoma of the orbit is a B-cell non-Hodgkin lymphoma and one of the most common orbital tumors, accounting for as much as half of all orbital malignancies. On imaging, orbital lymphoma usually appear as a soft tissue mass, often located in the upper outer quadrant in association with the lacrimal gland [2]. In distinguishing lymphomas from other orbital tumors, the extraocular muscles can be encircled or displaced. However, extraocular muscles are usually not the origin of the mass lesion. Infiltration of the optic nerve or eyeball is also rare. Similar to intracranial lymphomas they are homogeneous in density with high cellularity resulting in restricted diffusion on diffusion-weighted imaging (DWI), isointensity to hypointensity compared to muscle in T1-weighted sequences and isointense to hyperintense signal compared to muscle in T2-weighted imaging. After administration of Gd they show homogeneous enhancement [3, 4].

Orbital lymphoma seemed a valid differential diagnosis as the imaging criteria matched and lymphomas account for a large proportion of malignant orbital masses.

### Metastases

Orbital metastases are relatively uncommon with breast cancer being the most common malignancy to metastasize to the orbit, followed by prostate cancer, melanoma, and lung cancer [5]. Extraocular orbital metastases are usually unilateral and only rarely primarily involve the extraocular muscles, although secondary involvement may commonly occur [6]. Thyroid and prostate metastases can be located in the bony margins of the orbit. Radiographic features are variable both in computed tomography (CT) and MR imaging. The morphology ranges from well-defined to diffusely infiltrating lesions. Usually contrast enhancement is present but can be very variable. Bony destruction may be present. MR imaging shows its superiority compared to CT in greater contrast resolution making it invaluable in the assessment of orbital masses. Fat-suppression techniques and post-contrast T1-weighted images with thin slices and a reduced field of view are paramount for initial assessment [5, 7].

Although relatively uncommon in the orbit, metastases should always be considered as a differential diagnosis.

### Orbital Rhabdomyosarcoma

Rhabdomyosarcoma (RMS) is a highly malignant tumor. It has been reported from birth up to the seventh decade of life, with the majority of cases presenting in early childhood making it the most common soft tissue sarcoma of the head and neck in childhood. Orbital RMS is usually located extraconally or extending both intraconally and extraconally with close proximity to extraocular muscles. In early stages the tumor is usually well circumscribed, whereas in later stages borders become irregular [8]. On imaging, RMS are typically homogeneous soft tissue masses isodense to muscle and may show extension into the eyelid or through bony structures. MRI is the modality of choice for evaluating soft tissue tumors and plays an important role in initial diagnosis and assessment of tumor response after treatment [9]. RMS appear with low to intermediate intensity and isointense signal to adjacent muscles in T1-weighted sequences. They generally show vivid contrast enhancement. Because of high cellular density RMS usually have restricted diffusion on DWI [10, 11]. In terms of imaging features and tumor growth we considered RMS to be a possible diagnosis.

### Orbital Schwannoma

Schwannomas are benign nerve sheath tumors that originate from the Schwann cells of the perineurium of peripheral nerves. They are the most common benign peripheral nerve tumors in adults but rarely occur in the orbit [12]. Orbital schwannomas account for only 1% of all orbital tumors and commonly arise from supraorbital and supratrochlear nerves in the upper anterior orbital cavity [12, 13].

It is difficult to differentiate orbital schwannomas from other intraorbital tumors. They are homogeneous, elongated, and oval to spindle-shaped lesions with a density similar to extraocular muscles. General imaging features include cystic and fatty degeneration. In larger schwannomas cystic degeneration or hemorrhage may occur and calcifications are rare. CT has less diagnostic value but may show characteristic expansion into bone. On MR imaging, orbital schwannomas are usually hypointense in T1-weighted imaging and hyperintense on T2-weighted imaging. After administration of Gd schwannomas enhance, either homogeneously or heterogeneously [14, 15]. In our case, we considered orbital schwannoma a valid differential diagnosis based on its location and cystic and solid appearance with vivid Gd enhancement.

### Solitary Fibrous Tumor

Solitary fibrous tumors (SFT) are rare mesenchymal neoplasms which account for less than 2% of all soft tissue tumors [16]. They usually present as a solitary well-circumscribed mass located in intraconal and extraconal spaces of the orbit. The lesion may show calcifications and necrosis with high vascularization. Also remodeling of the adjacent bone may be seen in larger tumors [17]. Isointense to hypointense signal on T2-weighted images and vivid enhancement with probable washout pattern are the main MRI characteristics of orbital SFT. Internal hemorrhage, cysts or fibrosis are best demonstrated in T2-weighted sequences as well [18, 19]. Although a rare entity, if imaging criteria are met SFT may be included in the differential diagnosis of orbital soft tissue masses.

## Histology and Immunohistochemistry

**Fig. 4:**
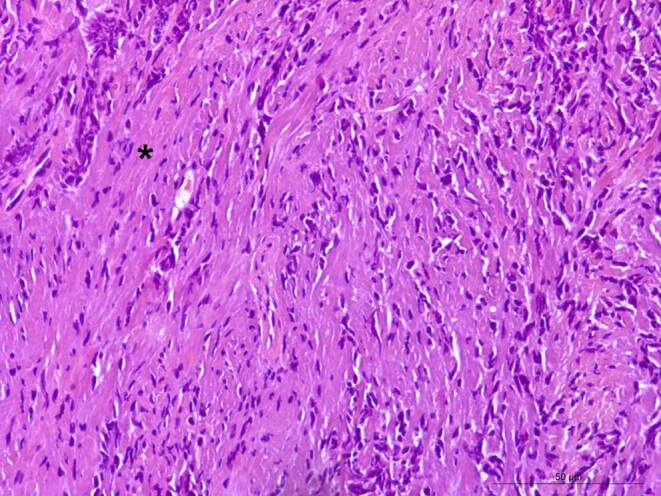
Hematoxylin-eosin-stained section depicted an isomorphic tumor formation with spindle-shaped cells and a streaming of tumor cells between collagen. *Asterisk* indicates collagenous stroma. Scale bar: 50 µm

**Fig. 5:**
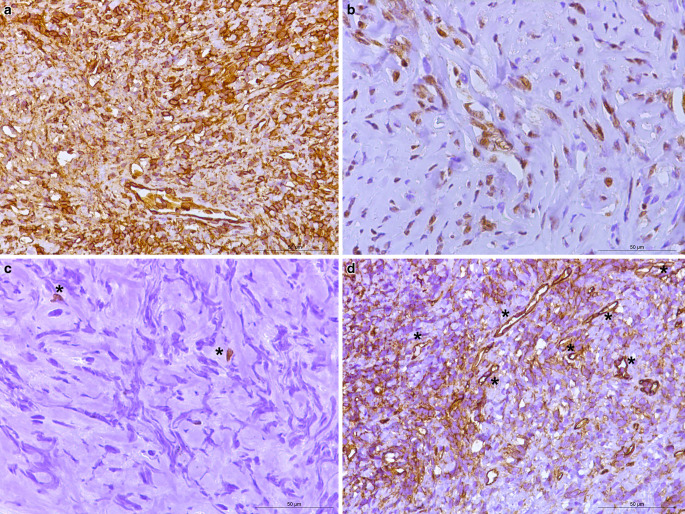
Immunohistochemistry: homogeneously positive reaction in the tumor cells for vimentin (**a**). Tumor cells showed a positive reaction for STAT6 (**b**). Immunohistochemistry for Ki-67 (Mib1), detected about 1% of all the tumor cells that were proliferating (**c**) with *asterisks* indicating two proliferating cells. Immunohistochemistry for CD34 (**d**) showed a positive reaction in the endothelial cells that constituted the dilated, branched, hyalinized staghorn-like (hemangiopericytoma-like) vasculature. *Asterisks* indicate a few exemplary vessels. Scale bars: 50 µm

In the hematoxylin-eosin (H&E) stained section of the formaldehyde-fixed and paraffin-embedded biopsy material, an isomorphic tumor was detected with moderately increased cellularity (Fig. 4). The tumor cells were mostly isomorphic and spindle-shaped. An increased number of blood vessels, and a collagenous stroma with streaming of cells between collagen was observed. No mitotic figures were identified. Fresh hemorrhages were present in a few, small regions. No traces of old hemorrhages were identified with the Prussian blue reaction (not shown). The tumor cells reacted positively in the immunohistochemistry for vimentin (Fig. 5a). In the immunohistochemistry for signal transducer and activator of transcription 6 (STAT6), a strong positive signal was observed in the nuclei of the tumor cells (Fig. 5b). The immunohistochemistry for inhibin, S100, pan-cytokeratin (PanCK), epithelial membrane antigen (EMA), and glucose transporter 1 (GLUT1) were negative in the tumor cells (not shown). The reaction for Ki-67 (Mib1) marked about 1% of all the tumor cells (Fig. 5c, asterisks). Numerous blood vessels (less so in number and intensity the tumor cells) were marked by the immunohistochemistry for CD34 (Fig. 5d, asterisk) and Wilm’s tumor protein (WT1, not shown). The positive immunohistochemistry for CD34 is characteristic (albeit nonspecific) for solitary fibrous tumors (SFT), especially low-grade SFT. The positive reaction for STAT6 in the tumor nuclei constitutes a very highly sensitive and specific marker for SFT [20]. About 98% of SFT cases have been described to show nuclear expression of STAT6, making it the most specific immmunohistochemical marker [21]. Nuclei positivity for STAT6 thereby reliably differentiates SFTs from meningioma, meningeal Ewingʼs sarcoma, mesenchymal chondrosarcoma, malignant peripheral nerve sheath tumor, and synovial sarcomas [20]. To further rule out the differential diagnosis of a malignant peripheral nerve sheath tumor, immunohistochemistry for S100 was performed, which produced a negative result (not shown). Likewise, immunohistochemistry was used to check for the presence of monophasic synovial sarcomas, again, yielding a negative result (not shown). The nuclei in this sample were oval but lacked the pseudoinclusions typical for meningioma. In addition, no calcifications or psammoma bodies were observed. Both observations ruling out the presence of a meningothelial neoplasm.

## Diagnosis

### Solitary fibrous tumor (SFT) of the orbit

Solitary fibrous tumors are rare mesenchymal tumors that arise at a plethora of anatomic sites, especially in deep soft tissues, and particularly in the thigh, pelvic fossa, retroperitoneum, and serosal surfaces [22–24]. The tumor cells carry a NAB2:STAT6 gene fusion, which is a result of a paracentric inversion involving chromosome 12q13 [20]. Orbital SFT appear mostly in middle-aged patients and are predominantly located in the superior aspect of the orbit [25]. Previous reports have concluded that orbital SFT are mostly benign tumors with a low recurrence rate of approximately 16% [26–29]. Surgical excision is the treatment of choice but can be difficult to achieve [25]. Head and neck solitary fibrous tumors demonstrate a significantly larger local recurrence rate as compared with the rate of metastasis. They can recur many years after initial treatment, warranting long-term surveillance and follow-up to assess for tumor recurrence [30]. Malignant SFT is extremely rare and it can be difficult to distinguish between benign and malignant SFT. Generally, malignant SFTs are larger than benign SFTs and common gross features are hemorrhage and/or necrosis in the malignant neoplasm [31]. Recent studies suggest that the presence of a telomerase reverse transcriptase (TERT) promoter mutation resulting in its overexpression may be associated with a shorter disease-free survival [32, 33].
